# Movements after Captive Bolt Stunning in Cattle and Possible Animal- and Process-Related Impact Factors—A Field Study

**DOI:** 10.3390/ani14071112

**Published:** 2024-04-04

**Authors:** Anika Lücking, Helen Louton, Martin von Wenzlawowicz, Michael Erhard, Karen von Holleben

**Affiliations:** 1 bsi Schwarzenbek—Training and Consultancy Institute for Animal Welfare at Transport and Slaughter, Grabauer Str 27A, 21493 Schwarzenbek, Germany; mvw@bsi-schwarzenbek.de (M.v.W.); info@bsi-schwarzenbek.de (K.v.H.); 2 Animal Health and Animal Welfare, Faculty of Agricultural and Environmental Sciences, University of Rostock, Justus-von-Liebig-Weg 6b, 18059 Rostock, Germany; helen.louton@uni-rostock.de; 3 Animal Welfare, Animal Behavior, Animal Hygiene and Animal Husbandry, Department of Veterinary Sciences, Faculty of Veterinary Medicine, LMU Munich, 80539 Munich, Germany; m.erhard@tierhyg.vetmed.uni-muenchen.de

**Keywords:** animal welfare, captive bolt, cattle, movements, slaughter, stunning effectiveness, unconsciousness

## Abstract

**Simple Summary:**

Regarding animal welfare during cattle slaughter, two issues emerge: firstly, consumers are becoming steadily alienated from meat production, and secondly, some media-released slaughterhouse footage is causing dissatisfaction among a public increasingly focused on animal welfare. Such footage often reveals cattle kicking during shackling and exsanguination, described by commentators as indicative of insufficient stunning effectiveness. Consequently, this undermines confidence in both the meat industry and the competence of supervising authorities. At slaughter, cattle movements after stunning affect occupational safety and often lead to a prolonged interval until bleeding occurs. The objective of this study was to comprehensively describe and analyse these movements in cattle (*Bos taurus*) in relation to stunning effectiveness, as well as to identify influencing factors. The results show that movements occurred in most cattle after captive bolt stunning. However, none of the movements observed were related to stunning effectiveness. Breed type and sex category, as well as the type and design of the captive bolt stunner used, influenced the movements of cattle after stunning. The results of this investigation underscore the importance of applying reliable indicators to assess stunning effectiveness.

**Abstract:**

Movements in cattle after captive bolt stunning cause problems in the slaughter process and lead to uncertainties in assessing stunning effectiveness. The objective of this study was to categorize and quantify these movements and determine animal- and process-related impact factors, as well as connections to stunning effectiveness and shooting position. In total 2911 cows, heifers, and bulls (dairy, beef, and crossbreeds) were examined (mean age 3.02 years). Movements from landing until at least four minutes after sticking were recorded by action cams (Apeman^®^ A100). Nine movement categories were defined (“kicking hind limb”, “twitching”, “bending and stretching hind limb”, “lifting and bending forelimb”, “body arching laterally”, “body arching ventrally”, and “arching backwards”). According to the movement severity, a score was assigned to each category. The scores were summed, either for certain process intervals, e.g., LANDING (ejection from the stunning box), HOISTING, or STICKING, or for the total time between LANDING and end of the FOURTH MINUTE OF BLEEDING (sum score). Statistical analysis (ANOVA) was performed on the scores. Only 6.6% of cattle showed no movement. Most movements occurred during STICKING and FIRST MINUTE OF BLEEDING, occurring rarely up to 8 min after sticking. While cows moved most at LANDING, bulls and heifers moved more if all process intervals were considered. The sum score was highest in German Angus, Charolais, and Limousin and lowest in Brown Swiss and Simmental. The score at LANDING was highest in German Angus and Black Holstein. The use of pneumatic stunners and an increase in bolt-exit length significantly reduced movements. No impact of stunning effectiveness on movements was found, but only 19 cattle showed reduced effectiveness.

## 1. Introduction

In the European Union (EU), most cattle are slaughtered after captive bolt stunning using pneumatic or cartridge-driven devices [1]. After stunning, the collapsed cattle are released from the restraining box onto a landing grid, where one hind leg is shackled, and the cattle are hoisted for sticking (exsanguination). In Germany, bleeding is usually performed via chest stick after a skin incision and changing of the knife.

According to Regulation (EC) No. 1099/2009 [2] on the protection of animals at the time of killing, restraining equipment and facilities should be designed and used to optimise the application of the stunning method, prevent injury or contusions to the animals, and minimize struggling. If cattle are stunned using pneumatic captive bolt devices, restraining boxes must restrict both the lateral and vertical movement of the animal’s head during stunning. The regulation requires effective stunning and comprehensive monitoring of stunning effectiveness to prevent the animal from being conscious during slaughter and subsequent exsanguination, experiencing pain, fear, and anxiety before finally dying due to blood loss. To ensure good stunning effectiveness, restraining, stunning, and bleeding must be carried out by persons holding a certificate of competence [2]. Each stunning method should adhere to defined key parameters, and the equipment utilized must be well maintained to ensure the effective stunning of all animals [3]. For penetrative captive bolt stunning, these key parameters include the position and direction of the shot, appropriate velocity, exit length and diameter of the bolt according to the animal’s size and species, and the maximum stun-to-stick/kill interval. In Germany, the Animal Welfare at Slaughter Ordinance specifies a maximum interval between stunning and sticking of 60 s for cattle [4].

Before 2001, the pithing rod was regularly used in cattle after captive bolt stunning, both to meet the time limit for bleeding and to reduce the reflex kicking movements. Pithing and, thus, mechanical destruction of the brain and spinal cord prevented the animal from regaining consciousness during exsanguination. By reducing post-stun movements at the same time, pithing also led to a lower risk of injury for staff at hoisting and sticking. Due to the increasing incidence of bovine spongiform encephalopathy (BSE) in Europe in the mid-1990s, from 1 January 2001 onwards the European Commission banned the use of pithing rods in cattle (*Bos taurus*), sheep (*Ovis gmelina aries*), and goats (*Capra aegagrus hircus*) slaughtered for human consumption [5]. Following this ban, convulsions and reflex-like body movements significantly increased in cattle after captive bolt stunning [6]. Furthermore, more animals regained consciousness during bleeding [7,8,9,10,11]. This was mostly a result of deficiencies regarding the shot placement, the stunning equipment used, and/or the strength of the cartridge used [6]. Both the increased occurrence of movements and signs of regaining consciousness during bleeding were likely to be due to the lack of pithing. However, due to the increasing use of modern stun boxes with tight head restraints and the development of more powerful captive bolt devices (especially pneumatically powered devices), stunning effectiveness at European cattle abattoirs has improved significantly in recent years [1,12]. This also applies to other bovines [13].

Previous studies indicate that following the ban on pithing, more than half of all captive bolt-stunned cattle show movements during hoisting, sticking, and bleeding [14,15]. However, movements do not always occur in a consistent pattern. Martin et al. [16] already found that Holstein cattle showed more kicking movements than other breeds, especially when stunned using a device with a relatively longer exit length of the bolt. In studies by Terlouw et al. [17], all cattle showed at least one movement after stunning, and post-stun movements by cattle with the spinal cord severed after stunning did not differ from those by cattle on which this manipulation was not performed. Clonic seizures such as reflex-like paddling of the hind limb are signs of a correctly performed bolt stun in cattle [17,18]. This is caused by the lack of inhibition of medullary or spinal reflexes due to the trauma-induced failure of higher-level centres in the brain [19] and also explains the occurrence of the above-mentioned convulsions during a zero-line EEG [20,21].

Nevertheless, there are currently two problems in practice since pithing has been banned. While the sometimes-strong excitatory movements often impede quick and safe shackling and sticking [22], leading to an extended stun-to-stick interval, there is also inherent uncertainty in the assessment of stunning effectiveness regarding movements. This uncertainty is heightened by the growing prevalence of video surveillance in abattoirs, coupled with public reactions to unauthorized video footage entering a society that is increasingly concerned about animal welfare. Consequently, the attention of animal welfare activists and some veterinarians is repeatedly drawn to the sometimes very impressive movements, which are then interpreted as a sign of regained consciousness. In the literature, there are only a few detailed descriptions of these post-stun movements and the possible impact factors, further contributing to existing uncertainty.

The aim of this publication is to record (1) the occurrence of movements after captive bolt stunning in cattle and to describe them in detail and to identify (2) animal-related and (3) process-related impact factors, as well as (4) a possible connection to reduced stunning effectiveness. The process-related factors included in the analysis also cover the various key parameters of the bolt gun. We hypothesised that movements would regularly occur in well-stunned cattle and that cows, especially the Black Holstein breed, would show the most movements. Furthermore, we hypothesised an association between the velocity of the bolt and the amount of movement. This work aims to contribute to the correct assessment of stunning effectiveness in cattle following captive bolt stunning.

## 2. Materials and Methods

### 2.1. Experimental Procedure

The investigation took place between June 2020 and April 2021 at five different German abattoirs (A–E), on two to four days each during routine slaughter. On each examination day, approximately 200 cattle were continuously observed by two pre-trained veterinarian investigators and filmed with the help of three to four action cameras, starting at stunning and lasting for at least four minutes after sticking. One camera was mounted above the restraining device, focussed on the animal’s head and body, to evaluate the effectiveness of the shot and detect second shots. The other cameras were positioned to continuously observe the head and body of the animals from landing (ejection from the stunning box) until at least four minutes after sticking. For each animal, animal-related and process-related factors were collected. The animal-related factors were sex category (bulls, heifers, and cows), breed, and carcass weight, as well as fat and conformation class, using the European Union’s EUROP grid method of beef carcass classification. The traditional grid is commonly used by most beef plants. Conformation is assessed on an E to P basis (EUROP), with E being a convex and shapely carcass, R being an average shape or straight profile, and P being a plainer carcass with a concave profile. Fat is assessed on a 1 to 5 basis, with 1 being very lean and 5 being very fat. Regarding stunning equipment, the device model of the stunner, function type (cartridge or pneumatic), cartridge strength (resp., air pressure) used, exit length of the bolt, bolt diameter, bolt velocity and weight, and the resulting kinetic energy were monitored. In addition, the stunner operator, the stun-to-stick interval, and the start of further dressing procedures (duration of bleeding) were recorded.

### 2.2. Slaughter Facilities

The participating abattoirs were medium to large-sized slaughter facilities with a daily slaughter capacity of 300 to 1000 animals (Table 1).

**Figure 1:**
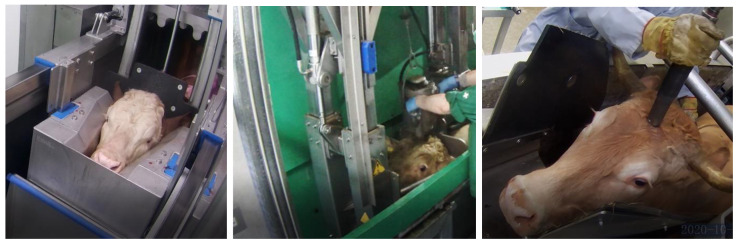
Different types of stun boxes to restrain cattle for captive bolt stunning; tight (active) head restraint DGS^®^ (**left**) and Banss^®^ (**middle**), and self-built loose (passive) head restraint (**right**).

At all facilities, cattle were delivered on the day of slaughter and temporarily (20 min to 7 h) kept in lairage. Except for abattoir E, all facilities used a modern stun box with tight head restraint (active) for restraining before stunning (Figure 1). The staff performing the stunning were trained (certificate of competence) and were aware of the optimum shooting position, which is 1.0 cm above the intersection of two imaginary lines between the centre of the eye and the opposite centre of the horn base, with deviations of less than 2 cm, and a shooting direction perpendicular to the skull [1].

### 2.3. Animals

A total of 2911 cattle, consisting of dairy, beef, and crossbreeds, were examined. Twenty animals were excluded from this study due to missing information, particularly due to incomplete video recordings. Further analyses thus included 2891 cattle. Of these, 31.4% were cows, 51.3% bulls, and 17.2% heifers. The distribution included 48.5% dairy cattle, 10.9% beef cattle, and 40.6% dual-purpose breeds. The most common breeds were Black Holstein (40.6%), Simmental (18.6%), and crossbreeds (14.7%). Most of the cows (79.0%) were Black Holstein. Among the bulls and heifers, the breeds Simmental (32.9%/8.0%), crossbreed (19.3%/24.3%), and Black Holstein (17.7%/39.0%) were predominant. The information on the breed, sex category, age, and carcass weight of the selected animals was gained from the cattle passports and slaughter lists.

### 2.4. Key Parameters and Features of Captive Bolt Devices

Different penetrating captive bolt devices, both pneumatic and cartridge-driven, and powered by different cartridge strength or air pressures, were used. For specifications, see Table 2.

All the devices used were examined beforehand. The diameter of each bolt was determined using a digital calliper (Digital ABS AOS calliper, Mitutoyo Germany GmbH, Neuss, Germany), and the weight of the bolt was determined using a precision balance (Sartorius ENTRIS II precision balance, WHI-Wägetechnik für Handel und Industrie GmbH & Co. KG, Hamburg, Germany). In order to obtain comparable values for exit velocity and kinetic energy, all stunning devices included were checked using a velocity tester (AST-106; AST 107-111 Stun Tester, Jarvis Products Corporation, Middletown, CT, USA, https://jarvisproducts.com/ accessed on 1 November 2023). The kinetic energy was then calculated from the velocity and bolt weight.

Ekin = ½ × m × v^2^

To determine the exit length, the stunning device used was shot at floral foam (ELES VIDA^®^, Bremen, Germany), and then the penetration depth was measured using a calliper. Measurements of the velocity and exit length were performed for all combinations of cartridge strengths or pneumatic pressure applied. To validate the method of exit length and velocity determination, a portion of the devices was rechecked in collaboration with a federal physics institute (PTB, Braunschweig, Germany). For this purpose, the shooting process was recorded using a high-speed camera (Fastcam 20,000 fps (frames per second), Photron, Reutlingen, Germany) and the recordings were then analysed using specific software (“Tracker”; https://physlets.org/tracker; open-source Physics, version number 5.1.5, accessed on 1 May 2020). The values determined by both measuring methods match well, although we could only perform the tests on 3–4 shots using a combination of cartridge and pressure.

### 2.5. Measurements

After the cattle had been stunned, an employee of the abattoir checked the state of consciousness in the stun box (collapse, relaxed eyelids, and ears) and at the landing grid (wide pupil, fixed eye, and no breathing; see Table 3) before they were shackled and hoisted. Bleeding was carried out via a chest stick after a previous skin incision (two-knife technique). Except at abattoir B, the chest stick procedure was performed using a single-edged slaughter knife. Abattoir B used a double-edged knife with a hollow handle and a peristaltic pump for blood collection. Individual animals were bled in a recumbent position if hoisting was delayed due to strong tonic and clonic seizures. The time to further dressing was between three and seven minutes. Of the two veterinarian investigators one had 30 years of experience and the other had trained for 10 months in advance to determine stunning effectiveness, respectively. An animal was considered properly stunned when it collapses immediately after stunning, exhibits tonic and clonic seizures, floppy ears, and no attempts to regain posture, both on the landing grid and during hoisting, sticking, and exsanguination. Additionally, apnoea starts immediately after the shot, corneal reflex on the landing grid is negative and throughout the entire observation period the eyes remain fixed with dilated pupils and no respiratory movements are noticed [23]. We distinguished between signs of sufficient stunning, doubtful stunning, and insufficient stunning (Table 3). Sufficiently stunned animals do not exhibit any signs of preserved brainstem activity, such as eye or eyelid movements and respiratory movements. The category insufficient stunning includes a high risk of regaining consciousness as well as remaining or regained consciousness [9].

**Figure 2:**
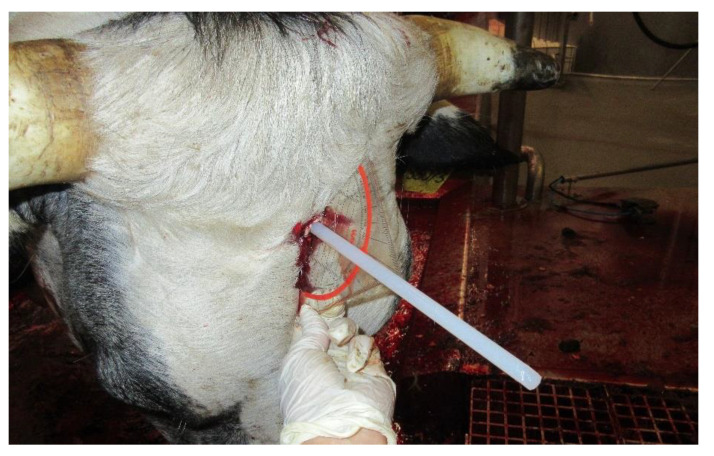
Recording possible deviations of shot position and angle using a plastic stick inserted in the shot hole and a geo-triangle at the end of the bleeding line.

The first investigator stood next to the head of the animal at the landing grid and recorded the ear tag number, stunning efficiency during LANDING and HOISTING, and stun-to-stick interval (stopwatch; Delta E 100, Hanhart 1882 GmbH. Gütenbach, Germany). The second investigator checked stunning efficiency during bleeding and subsequently measured the angle and position of the shot hole using a geo-triangle (Westcott E-10132 00 Geodreieck, Acme United Europe GmbH, Solingen, Germany), a plastic stick (diameter 8.4 mm or 12.4 mm, depending on the bolt diameter; POM round bar, Nattmann GmbH, Willich, Germany; Figure 2), and a multi-angle ruler (Wohao, Shenzen, China; Figure 2). Deviations of at least 2 cm from the ideal shooting position [1] and at least 10° from the perpendicular were recorded.

On each examination day, three to four action cameras (Apeman A100S, Apeman, Shenzhen, China, https://de.apemans.com/collections/action-kamera, accessed on 1 November 2023) were installed, depending on the facility, to record each animal from the stun box until at least the end of the fourth minute after sticking. The observation period was divided into eight process intervals. The first interval, LANDING (1), started when the animal fell out of the stun box and touched the landing grid (approx. 7 s–20 s after the shot) and ended as soon as the shackle chain tightened during lifting (approx. 15 s–55 s after shot). The next process interval, HOISTING (2), was followed by SKIN INCISION (3). The latter started with the first contact between knife and skin (approx. 24 s–60 s after the shot) and ended before STICKING via the chest stick (4). This interval started when the knife was inserted into the chest entrance (26 s–78 s after the shot) and ended 3 s after the knife had been pulled out. STICKING was followed by the FIRST (5), SECOND (6), THIRD (7), and FOURTH MINUTE OF BLEEDING (8). These last four process intervals each lasted for one minute, the first starting four seconds after STICKING and the last ending 240 s later at the earliest (maximum 400 s). The subsequent analysis of the video material regarding movement category, frequency, and time of movement was always carried out by the same pre-trained investigator. For each animal, the movement categories were recorded based on process interval. Each movement category was counted only once per process interval. A score value was assigned to each movement category (0–3), reflecting the severity of the movement in terms of vigour, speed, frequency, and impact on the process. A score per animal and process interval was obtained by addition. The scores of all process intervals summed up resulted in the total score (sum score) for an individual animal. The definitions of movement categories and scores are shown in Table 4. Examples showing the different movement categories can be found as movies in the Supplementary Materials.

### 2.6. Statistical Analysis

Data were documented in MS Excel (Microsoft Corporation, (2018), Microsoft Excel, Redmond, WA, USA) and pivot tables were used for plotting and analysis. The software JMP v. 15 (SAS Institute Inc., Cary, NC, USA) was used for statistical analyses. The dependent variable was the sum score across all process intervals or the score of a specific process interval, calculated for each individual animal. The influence of possible animal- and process-related factors (independent variables) on the scores was investigated via analysis of variance (ANOVA model) and the Tukey–Kramer post hoc test to compare means. A result was considered statistically significant for *p* < 0.05. Independent variables included in the model were captive bolt parameters, model and function type of stunning device (pneumatic or cartridge powered), exit length, diameter, velocity, and kinetic energy of the bolt. Animal-related factors included were sex category (cow, heifer, and bull), breed, carcass weight, fat, and conformation class. Other variables in the model were stunning effectiveness, as well as the process-related variables stun-to-stick interval and deviation in position or angle of the shot. The relative frequencies of individual nominal parameters were described using contingency tables and tested for random distribution using Chi-square tests. In addition, decision trees (stepwise partitions) were used to determine combinations (of independent variables) that most strongly influence a certain expression of the sum score. In this process, the sample is split into subgroups in such a way that the means of the sum score of subgroups differ as much as possible until no further sensible differentiation can be made. Only effects leading to significant deviations from the mean are presented, and results are not shown when concerning an insignificantly small group (threshold: n < 146 ≙ 5%).

## 3. Results

### 3.1. Occurrence of Movements

Most cattle showed movements after captive bolt stunning (number of movements across all process intervals: minimum: 0; maximum: 15; mean: 3.5). Only 6.6% of the animals did not show any movement at any process interval (bulls 6.1%, heifers 4.0%, cows 8.7%). Most movements were recorded in the process interval FIRST MINUTE OF BLEEDING. Here, 61.7% of the animals showed at least one movement. In addition, at STICKING, more than half of the cattle (58.0%) showed at least one movement. In the SECOND (14.2%) and THIRD MINUTES (6.4%) OF BLEEDING, considerably fewer movements were observed in cattle compared with the FIRST MINUTE OF BLEEDING. In the FOURTH MINUTE OF BLEEDING, the proportion of animals moving increased slightly (12.2%). Late movements during bleeding also occurred in cattle that had previously been hanging on the rail completely motionless and with limp tails. In some cattle, movements could still be observed more than 8 min after STICKING. During one process interval, several movements of different categories could occur. In some animals, up to five different movement categories were observed within the same process interval.

**Figure 3:**
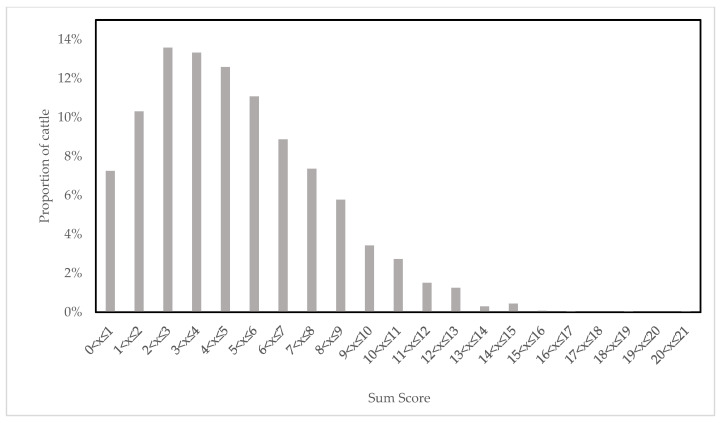
Distribution of sum score values (a measure of frequency and intensity of an animal’s movements across all process intervals; mean: 4.87; minimum: 0; maximum: 20.5; and median: 4.5) in the study group (N = 2891) (relative frequency).

The mean sum score, a measure of the frequency and intensity of an animal’s movements across all process intervals, was 4.87 (minimum: 0; maximum: 20.5; median: 4.5). The interquartile range of the sum score was 2.5 to 7.0. The distribution of the sum score is shown in Figure 3. The mean score at LANDING was 0.79 (minimum: 0; maximum: 6.0; median: 0).

All movements occurred spontaneously as well as in connection with manipulation (grasping and lifting limbs, pulling of the shackle on the hind limb, sticking, and further dressing of the lower feet). Particularly strong and intense movements were observed at LANDING and during HOISTING. Movements were obvious during LANDING in 31.7% of the cattle and during HOISTING in 43.7%. The most common movements during these early process intervals were “kicking hind limb moderate” (21.9%), “twitching moderate” (18.0%), and “twitching intense” (13.6%). In particular, “kicking hind limb intense” and “twitching intense” on the landing grid made it very difficult for the staff to start up shackling, which often led to an extended stun-to-stick interval. Occasionally, the “kicking hind limb” and “twitching” movements caused shackles to be thrown out of position. This was especially observed in Holstein dairy cows.

During the process intervals SKIN INCISION and STICKING, “kicking hind limb” (39.0%), “body arching ventrally” (25.5%), “lifting forelimb” (22.9%) and “bending forelimb” (22.4%) were observed most frequently. The majority of late movements towards the end of exsanguination were “stretching hind limb” (7.2%), “kicking hind limb” (3.7%), and “bending forelimb” (1.1%). In the late phases of bleeding, it was frequently noticed that the tail tension temporarily increased again. In some cases, there was also intensive tail flapping, even though the tail had already been hanging limply for minutes before. None of the animals that moved for more than four minutes after sticking showed signs of regaining consciousness.

#### Category of Movements during Different Process Intervals

**Figure 4:**
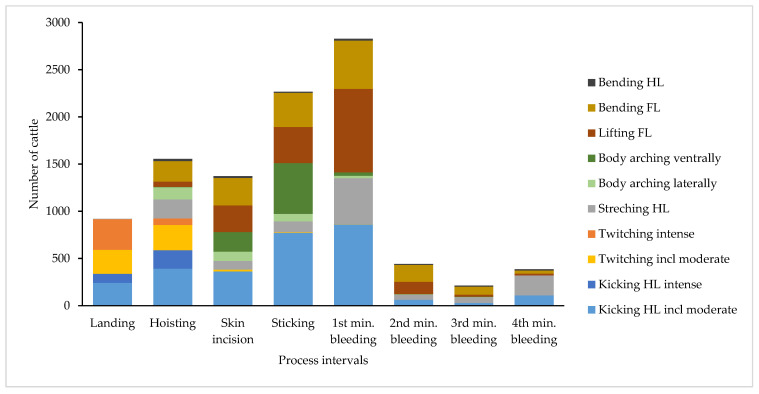
The absolute number of observed movement categories related to the eight different process intervals (more than one movement by animal and process interval possible); HL = hind limb; FL = forelimb.

The absolute number of individual movements related to the eight different process intervals is shown in Figure 4. The movement most frequently counted was “kicking hind limb”, including “kicking hind limb moderate” (n = 2810). Kicking occurred in each process interval but most frequently between LANDING and the end of the FIRST MINUTE OF BLEEDING. In some cases, “kicking hind limb” was observed over 50 times, lasting several process intervals. During LANDING and HOISTING, 21.9% of the animals expressed “kicking hind limb moderate” and 10.0% expressed “kicking hind limb intense”. During HOISTING, “kicking hind limb”, either moderate or intense, was observed in 20.3% of the cattle (18.8% bulls, 27.7% heifers, 18.7% cows). “Kicking hind limb” during STICKING occurred in 41.6% of the animals (32.0% bulls, 30.0% heifers, 63.0% cows). The next frequent movements were “lifting forelimb” (n = 1761) and “bending forelimb” (n = 1655), which occurred in all process intervals from HOISTING onwards but most frequently during SKIN INCISION, STICKING, and the FIRST MINUTE OF BLEEDING. We observed that “bending forelimb” tended to occur later than “lifting forelimb”. “Twitching”, either moderate or intense, occurred almost exclusively during LANDING or HOISTING. “Stretching hind limb” was observed in each process interval, but most frequently in the FIRST MINUTE OF BLEEDING. The slight increase in movements at the FOURTH MINUTE OF BLEEDING was predominantly due to “stretching hind limb”, often together with shivering of the same. The category “body arching ventrally” almost always occurred during SKIN INCISION or STICKING, and in individual cases, also during HOISTING or still in the FIRST MINUTE OF BLEEDING (see Figure 4). In the later course of bleeding (SECOND MINUTE OF BLEEDING and later), “body arching ventrally” was observed only four times. In particular, provoked by sticking, cattle moved their heads and necks vigorously in the ventral direction. Across all process intervals, “body arching ventrally” was observed in 649 cattle (22.4%), regularly accompanied by “kicking hind limb” or “lifting forelimb”. “Body arching laterally” was detected in 291 animals overall (10.0%), mainly during HOISTING, SKIN INCISION, and STICKING. Only six times was this category of movement observed at a later process interval, i.e., four times during the SECOND MINUTE and twice during the THIRD MINUTE OF BLEEDING. The movement of “body arching laterally” as such could either be short or held for more than a minute. In this study, no “arching backwards” was noticed in any animal.

### 3.2. The Effect of Animal-Related Factors on Movements

#### 3.2.1. The Effect of Sex Category

**Figure 5:**
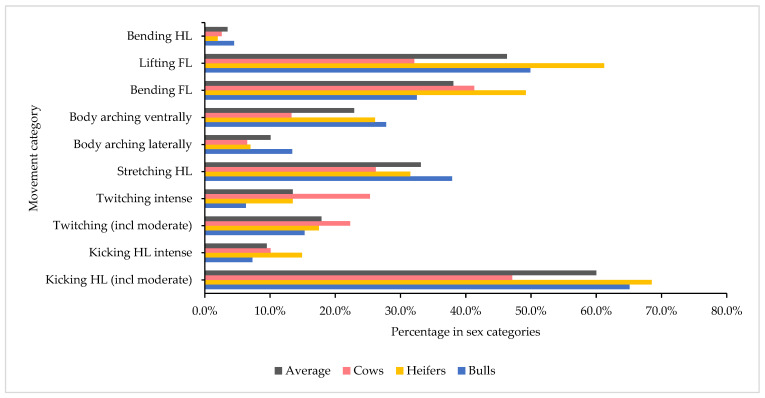
Relative frequency of the movement categories across all process intervals in relation to sex categories (cows, heifers, and bulls); HL = hind limb; FL = forelimb.

Sex categories differed in terms of frequency of movement during the different process intervals. A total of 48.9% of cows, 37.2% of heifers, and 19.1% of bulls showed movements at LANDING. During HOISTING, the percentage of heifers and bulls with movements increased (52.4% and 39.7%, respectively), while the percentage of cows decreased (45.4%). During SKIN INCISION and STICKING, most heifers (78.3%) and bulls (70.3%) showed at least one movement, whereas this was the case for less than half of the cows (47.2%). During the four-minute bleeding interval, 78.3% of heifers, 70.3% of bulls, and 62.3% of cows still showed at least one movement. In addition, concerning the frequency of movement categories, differences between sex categories were determined for certain categories. Both “twitching moderate” and “twitching intense” were observed, especially in cows (see Figure 5). According to the ANOVA, “body arching laterally” and “body arching ventrally” occurred significantly more in bulls and less in cows (*p* < 0.001). Forelimb movements occurred significantly more in heifers (*p* < 0.001).

The results of the contingency test comparing the sex categories regarding movement frequencies by process intervals show that, especially at LANDING (*p* < 0.001), “twitching intense” occurred significantly more often in cows (22.8%) than in heifers (11.2%) or bulls (4.1%). At LANDING (*p* < 0.001) and HOISTING (*p* < 0.001), “kicking hind limb intense” was observed significantly more often in heifers (5.6%/11.2%) than in bulls (1.5%/5.9%). At STICKING (*p* < 0.001), “body arching ventrally” was recorded significantly more often for heifers (22.9%) and bulls (21.6%) than for cows (10.6%). “Lifting forelimb” in the FIRST MINUTE OF BLEEDING (*p* < 0.001) was observed significantly more often in heifers (42.4%) and bulls (33.2%) than in cows (18.7%).

Heifers had the highest average sum score. Despite the intensive movements at LANDING, across all process intervals cows had a lower average sum score (mean: 4.42) than heifers (mean: 6.06) or bulls (mean: 4.70). The ANOVA revealed a significant effect of sex category on the sum score (*p* < 0.0001), with least square means being significantly lower in cows (5.58) than in bulls (6.74) and heifers (6.90). However, in the ANOVAs used to check animal- and process-related impacts on scores, the variables examined explained only 10.0% to 12.0% of the score variability.

#### 3.2.2. The Effect of Breed

Table 5 displays the frequency of movement categories for the most commonly represented breed types. Some breeds differed considerably regarding the category of movements and frequency of occurrence. “Twitching intense” during LANDING or HOISTING was shown especially by German Angus (GA: 21.4%, total: 13.5%), Black Holstein (20.6%), Red Holstein (19.4%), and Limousin (18.9%) but rarely by Brown Swiss (0.0%) and Simmental (3.5%). “Kicking hind limb intense” during LANDING and HOISTING was observed most frequently in Limousin (LIM: 16.7%, total: 9.6%) and Red Holstein (16.0%) and least frequently in Brown Swiss (2.0%). “Stretching hind limb” occurred particularly often in Limousin (LIM: 50.0%, total: 32.9%), and “body arching ventrally” occurred in German Angus and Charolais (see Table 5).

The highest average sum score was measured for German Angus (mean: 6.46), followed by Charolais (mean: 6.04) and Limousin (mean: 5.91). Breeds with low sum scores covered Brown Swiss (3.21) and Simmental (mean: 3.88). The score at LANDING was highest in German Angus (mean: 1.29), Black Holstein (mean: 1.08), and Red Holstein (mean: 1.01).

The ANOVA revealed a significant impact of the breed type (*p* < 0.0001) on the sum score. The breeds Brown Swiss and Simmental significantly decreased, while German Angus increased the sum score. Least square means for German Angus (7.84), Charolais (7.35), Limousin (6.75), Black Holstein (6.52), Crossbreed Beef × Beef (6.43), Crossbreed Beef × Dairy (6.39), and Red Holstein (6.22) were significantly higher than for Simmental (4.98) and Brown Swiss (4.21).

An impact of the class of conformation (E-P) on the extent of movements was found regarding the score at HOISTING (*p* < 0.01), which was higher in cattle classified as moderately conformed (R-O). Class of conformation also affected the score at STICKING (*p* < 0.01). Here, low conformation (O-P) decreased the score. The effect of slaughter weight was less significant (*p* < 0.05). In the model (ANOVA), an increase in slaughter weight by 1.0 kg each lowered the sum score by 0.003 (mean: 4.87).

### 3.3. The Effect of Process-Related Factors on Movements

Of the process-related factors, only those related to stunning devices are presented here. The ANOVA did not show an effect of the stun-to-stick interval (mean: 44.6 s, min: 26.0 s, and max: 78.0 s).

#### The Effect of Captive Bolt-Related Factors

Of 2891 animals examined, 731 (bulls n = 203, heifers n = 239, and cows n = 289) were stunned with cartridge-powered captive bolt devices (KS Schermer^®^ n = 301, KR Schermer^®^ n = 325, KL Schermer^®^ n = 105). The other 2160 animals (bulls n = 1281, heifers n = 259, and cows n = 620) were stunned with a pneumatically powered device (USSS-21 Jarvis^®^ n = 508, VB 315 EFA^®^ n = 1652). Overall, more movements were observed in cattle stunned with a cartridge-powered bolt gun than in cattle stunned with a pneumatic device. “Kicking hind limb intense” during LANDING and HOISTING, for example, occurred considerably more often in animals stunned with a cartridge-driven captive bolt (17.9%) than in animals stunned with a pneumatically powered device (6.6%). When comparing the two function types regarding movement frequencies by process intervals by means of contingency tests, i.e., without taking other effects into account (see Table 6), the described effect (cartridge-powered > pneumatic powered) often became significant (*p* < 0.001) for most movement categories and for the process intervals from HOISTING up to and including the FIRST MINUTE OF BLEEDING. An exception was “twitching moderate” at LANDING and HOISTING, which was observed more frequently for pneumatic guns (Table 6). However, most of the other movement categories, such as “kicking hind limb intense” at LANDING and HOISTING, “kicking hind limb moderate” at LANDING and HOISTING, “kicking hind limb” in the FIRST MINUTE OF BLEEDING, and “lifting forelimb” from HOISTING to FIRST MINUTE OF BLEEDING, also occurred significantly more often in animals stunned with a cartridge-powered device.

The average sum score was 5.68 for the cartridge-powered devices and 4.60 for the pneumatically powered devices. In particular, the greatest difference occurred between the pneumatically powered device VB-315 (mean: 4.34) and the two cartridge-powered devices KL (mean: 6.52) and KS (mean: 6.06).

The ANOVA revealed a significant impact of the function type (*p* < 0.001) on the sum score. In the model, the use of cartridge-powered stunners increased the sum score while the use of pneumatically powered stunners decreased it by 4.1. The exit length of the bolt had a decreasing impact on the frequency and intensity of movements (ANOVA, *p* < 0.001). In the statistical model, an increase in exit length by 1 mm reduced the sum score (mean: 4.87) by 0.044, and the score at LANDING (mean: 0.78) by 0.009. The effect of the bolt diameter was less clear (*p* < 0.05). In the model (ANOVA), increasing the bolt diameter by 1 mm increased the sum score (mean: 4.87) by 0.38 and the score at LANDING (mean: 0.78) by 0.26. The exit velocity of the bolt had an increasing effect on movements (*p* < 0.001). In the model (ANOVA), an increase in bolt velocity by 1 m/s increased the sum score by 0.07 and the score at LANDING by 0.03. This effect was only found if the function type (pneumatic or cartridge) and type of stunning device were not included in the analysis, as the effect was otherwise attributed to the function or type of device. A clear effect of bolt kinetic energy could not be shown by the ANOVA. An analysis of the decision trees revealed a lower sum score (mean: 4.43) for stunning devices with kinetic energy above 453 J (see Table 2) compared with devices with kinetic energy below 453 J (mean: 5.75). A similar effect was also found for the score at LANDING. Bulls stunned using devices of at least 488 J had a lower sum score at LANDING (mean: 0.32) than those stunned using devices with kinetic energy below 488 J (mean: 0.72).

### 3.4. The Effect of Stunning Quality on Movements

A total of 99.4% of the animals examined (n = 2911) were described as “sufficiently stunned” (bulls 99.5%, heifers 99.0%, and cows 99.3%). The stunning effect was stated as “doubtful” for 0.3% (n = 9: 3 bulls, 3 heifers, and 3 cows) and “insufficient” for 0.3% (n = 10: 5 bulls, 2 heifers, and 3 cows), of which only four cattle stayed conscious for a few seconds. Signs observed in animals called “doubtful” were twofold breathing movements (recognised on muzzle or nostrils), nystagmus at the landing grid, and returning tension/movement of the tongue. Animals classified as “insufficiently” stunned showed repeated respiratory movements (n > 4) at the muzzle or nostrils, spontaneous eyelid closure, or lack of collapse following the shot together with focused eye movements. In one animal showing multiple respiratory movements of muzzle and jaw, as well as a tense eyelid during bleeding, ear tension was regained three minutes after sticking. In two other animals that showed repeated respiratory movements and/or tongue tone, persistent “body arching laterally” was observed as well, lasting until the THIRD MINUTE OF BLEEDING. These two animals were the only ones that still showed “body arching laterally” after the end of the SECOND MINUTE OF BLEEDING. A total of 17 animals were re-stunned by the staff. All animals classified as “insufficiently” stunned (n = 10) and most of the animals classified as “doubtful” were immediately re-stunned on the spot by the staff. After case-by-case analysis, the following potential causes were considered. In 6 of 19 animals with “doubtful” or “insufficient” stunning effects, angular and/or positional deviation of the shot position of more than 20° or more than 3 cm were recorded. In two cattle, slight angular deviations (≤15°) were found together with slight deviations in shot position (≤2 cm). One animal had an abnormal swelling of the os frontale. In four other cases, it was suspected that the stunning device used (Schermer KS, Freund Maschinenfabrik GmbH & Co. KG, Paderborn, Germany) might not have been strong enough in relation to body weight. For the remaining six cases, no possible reason for reduced effectiveness could be identified. Overall (n = 2911), deviations in shooting position (≥2 cm from the ideal position) and/or angle (≥10°) were found in 21.1% (n = 614) of the cattle and we obtained indications for thereby increasing effects on movements. The sum score increased with increasing distance from the ideal shot position (<2 cm: mean 4.81; ≥2 cm: mean 5.46; ≥3 cm: mean 5.39; and ≥4 cm: mean 6.0). In the model (ANOVA), increasing the shot deviation by 1 cm increased the score at HOISTING (mean: 0.90) by 0.11 (*p* < 0.001). An increase in the sum score was also seen with angular deviations of more than 20° (mean: 5.35) compared with animals with angular deviations of less than 10° (mean: 4.85).

Due to the very small number of animals with the stunning effect stated as “doubtful” or “insufficient” (n = 19), no statistics are possible regarding the impact of stunning effectiveness on the sum score or the occurrence of certain movement categories. Proceeding purely descriptively, the sum score of the cattle categorised as “doubtful” (mean: 6.44, minimum: 3.00, and maximum: 10.00) or “insufficient” (mean: 5.90, minimum: 1.00, and maximum: 12.50) is comparable to the entire study group (mean: 4.87, minimum: 0.00, and maximum: 20.50). All movements observed during this investigation occurred in both well-stunned cattle and cattle with reduced stunning effectiveness.

## 4. Discussion

### 4.1. Occurrence of Movements

In this study, the analyses of movements show that after captive bolt stunning, there were hardly any cattle that did not show any movements at all. Overall, movements were observed in 93.4% of all cattle during at least one process interval. Most movements occurred during sticking (58.0%) and during the first minute of bleeding (61.7%). This could be attributed to the continued seizure activity after the shot [10,27] and the simultaneous reduction of inhibitory effects by higher brain centres already damaged by the effect of the captive bolt stun [28], while major manipulations such as hoisting and sticking could act as external triggers. This is in line with the results of Hilsenbeck [14] and those of von Holleben and von Wenzlawowicz [15]. Both studies report the proportion of cattle with movements during hoisting, sticking, and bleeding between 51.0% and 65.0%. In our study, a significant proportion of animals (12.2%; between 9.1% (bulls) and 14.4% (cows)) were found to still show movements in the fourth minute of bleeding. As well as described by Hilsenbeck [14] we observed that “stretching hind limb”, often in combination with shivering, was the most frequent movement during advanced exsanguination. Before these late movements occurred, cattle were usually already hanging completely relaxed with their tails hanging limply. This was also the case in one animal that was moving eight minutes after sticking. Our results show that all movements, including neck and back movements such as “body arching ventrally” (22.4%) and “body arching laterally” (10.0%), regularly occur in unconscious cattle after captive bolt stunning. In practice, however, the latter movements are often equated with “arching backwards”. Nevertheless, only “arching backwards” is a form of righting reflex [25,29], an active attempt of the animal to bring the head and body into a normal position, requiring a functional medulla and midbrain [30] and thus indicating the return of consciousness. “Body arching ventrally” in this study and in that by Terlouw et al. [17] was observed predominantly during sticking. In almost all cases, it occurred as a reflex-like response to the cutting of skin, muscles, and blood vessels. The fact that this movement was only very rarely observed without previous manipulation, e.g., during exsanguination, again confirms the trigger effect of manipulation. In this study, “body arching ventrally” was observed only four times during the second to third minute of bleeding, mainly at abattoir B, which was the only facility using a sticking knife with a hollow handle for blood collection. The late occurrence of “body arching ventrally” at this abattoir during bleeding could, therefore, be a consequence of the manipulation when removing the sticking knife. “Body arching laterally” in our study was observed predominantly during hoisting and in connection with the tonic phase, a typical sign for well-stunned animals after captive bolt stunning, during which the muscles of the back and legs are rigid and the hind limbs are flexed [27]. These clonic–tonic seizures typically occur following captive bolt stunning and may vary in duration [31]. Due to the use of modern stun boxes, nowadays cattle are often already shackled and hoisted during the tonic phase, which fosters the occurrence of “body arching laterally” [15]. In only two animals was “body arching laterally” observed during the third minute of bleeding.

### 4.2. Animal-Related Impact Factors on Movements

Our analyses demonstrate that the frequency of movements varies with regard to both sex category and breed. In particular, German Angus, Black Holstein, and Red Holstein exhibited a high movement score during landing, which may impede shackling. We confirmed the field experience that cows show especially strong movements such as “kicking hind limb intense” at landing. However, across all process intervals, cows moved less (mean sum score: 5.58) than heifers or bulls (mean sum score: 6.90 and 6.74, respectively), as from hoisting onwards they already showed less frequent movements. Our results are similar to those of von Holleben and von Wenzlawowicz [15], who recorded movements in 55.0% of bulls, 61.0% of heifers, and 51.0% of cows. Differences between sex categories regarding the frequency of certain movements were another result of our investigation, e.g., 30.6% of the bulls showed “body arching ventrally”, while this movement was only observed in 22.7% of cows. The latter, on the other hand, showed “twitching intense” significantly more often (23.2%) than did heifers (13.7%) or bulls (10.4%). By contrast, Terlouw et al. [17] did not find any clear differences between sex categories regarding type and frequency of movements, possibly because of the significantly smaller sample size (n = 40) in their study. Our results also differ compared with those of Hilsenbeck [14] regarding the frequency of “kicking hind limb” during hoisting, which was observed in 55.7% of cattle, while our results show a prevalence of 20.3% in cattle (18.8% bulls, 27.7% heifers, 18.7% cows). This could be attributed to the different groups of animals investigated or the exclusive use of cartridge-powered stunners in the study by Hilsenbeck [14]. However, the results are similar with regard to movements at sticking, with 34.3% of the cattle not showing any movements in Hilsenbeck [14], compared with 41.6% in the present study. Furthermore, we observed an impact of breed type on the sum score, as well as on the occurrence of certain movement categories. During hoisting, “kicking hind limb” was observed significantly more often in dairy cattle (e.g., Black Holstein) than in beef or cross breeds. Similar results were obtained by Martin et al. [16]. Results by Kline et al. [22] and von Holleben and von Wenzlawowicz [15] show that, especially during landing, strong movements like “twitching intense” and “kicking hind limb intense” occur more often in dairy breeds (Black and Red Holstein). Despite the high score for dairy breeds during hoisting and especially landing, we calculated a higher sum score for breeds like German Angus than for Black or Red Holstein. This is due to the finding that movements in German Angus lasted longer and, therefore, occurred over more process intervals than those in dairy breeds. A lower sum score than that for German Angus (mean: 6.46), Limousin (mean: 5.91), or Charolais (mean: 6.04) was obtained for Brown Swiss cattle (mean: 3.21). Skull anatomy features may have influenced the extent of movements, but our data do not provide conclusive evidence. For instance, Simmental and German Angus bulls share comparable skull anatomy but their sum scores differ significantly. A possible impact factor could be the animals’ temperament or the state of excitement/stress level just before stunning. Both physical exertion and pre-slaughter stress cause an increased muscle metabolism shortly after death [32] and could, therefore, affect the expression of spinal cord reflexes and automatisms. According to Grandin and Deesing [33], certain “Common Continental European breeds”, such as Limousin and Charolais, are known to be nervous and flighty. The observations made during our study indicate that temperament and stress impact could be important, without these two parameters explicitly being monitored.

### 4.3. Captive Bolt-Related Impact Factors on Movements

The effects of features of the stunning devices on movements cannot always be completely separated from each other. Our research showed that cattle stunned using pneumatically powered devices (mean sum score: 4.60) moved less than those stunned using cartridge-powered guns (mean sum score: 5.68). One possible reason for this could be the higher bolt velocity of cartridge-powered stunners. Pneumatically powered devices also differ from cartridge-powered devices in terms of their kinetic energy. The mass of the bolt is significantly higher for pneumatically powered guns, which, despite a lower bolt speed, results in higher kinetic energy than for cartridge-powered stunners. According to our study (analyses of decision trees), for a higher kinetic energy (>453 J), the average sum score—a measure of the frequency and intensity of movements—was lower. It is possible that, due to their higher kinetic energy, pneumatically powered devices caused more damage to the brain, especially the brainstem and upper spinal cord, thus reducing the occurrence of medullary and spinal reflexes. However, another reason for the more extensive damage in pneumatically stunned animals could be the use of a closer head restraint, which is usually practised when using pneumatic guns. As there is hardly any space left for the head to move while being shot, energy is transferred directly to the skull and brain without loss. By contrast, if hand-held cartridge-powered stunners are used, the heads of the animals are often not restrained as tightly, and kinetic energy may be lost if the head swerves on impact. The assumption that more pronounced damage in deep brain structures may reduce movements is also supported by the fact that in our analyses, an increasing exit length of the bolt was associated with a significantly lower sum score or score at hoisting. A longer bolt penetrates deeper into the brain, thus causing more extensive damage to deeper brain structures. However, our results regarding exit length are not in line with those by Martin et al. [16], who observed more “kicking hind limb” with increasing penetration depth in Holstein cattle. Martin et al. [16], however, considered just “kicking hind limb” and only in the period from hoisting to sticking, which may explain the deviating results, along with differences in the group of animals studied, captive bolt devices used, and the lack of head restraint when using a centre track conveyor restrainer system.

### 4.4. Stunning Quality

In this study, a “doubtful” or “insufficient” stunning effectiveness was found in only 0.6% of the cattle. This is a remarkable improvement compared with previous studies. For the period 2003 to 2012, rates of failed stunning were reported at 4.0% to 9.2% [7,8,9,10,11,34]. Dörfler [12] estimated the proportion of inadequately stunned cattle to be only between 0.9% and 1.9%, rising to 5.7% in exceptional cases. This trend is presumably due to the further development and improved maintenance of captive bolt stunning devices [35], the establishment of modern stun boxes with tight head restraints, and increasing animal welfare monitoring at abattoirs. The presence of the investigators may also have positively affected the performance of the staff performing the stunning. When using well-maintained modern captive bolt devices, slight deviations in position or angle do not necessarily lead to a reduced stunning effect [36,37], which is also confirmed by our results, as we found deviations in position or angle in 614 (21.1%) heads but only 19 out of 2911 showed signs of a reduced stunning effect. However, our results, as well as those of Ilgert [38] and Kaegi [39], indicate that, independently of stunning effectiveness, an increased deviation in shooting position or angle may lead to more movements.

Our results demonstrate that movements occur regularly in cattle that simultaneously show no signs of an active brainstem such as eye or respiratory movements. This is in line with previous studies, such as the work of Fricker and Riek [20], who confirmed that convulsive activity still occurs in cattle with absent brain function (isoelectric EEG). Convulsions result from the failure of higher-level motor control centres in the brain, thus being incompatible with a simultaneously maintained consciousness [18,28,40]. Therefore, the mere presence of movements is not suitable to distinguish between consciousness and unconsciousness [29]. Concerning “arching backwards”, which in cattle always indicates an insufficient stunning effect [25], studies by McKinstry and Anil [41] and Grandin [42] show that cattle when regaining consciousness first show a resumption of respiration or a positive corneal reflex before exhibiting righting reflex and attempts to raise their heads.

In this study, none of the animals showed “arching backwards”. With regard to “body arching laterally”, another movement often mistaken for the righting reflex, our investigation revealed that this movement occurs in 10.0% (n = 291) of all animals, mainly during hoisting and sticking, but in nearly all cases within two minutes after sticking. At this time, the animal may still show tonic–clonic seizures following the stun. In only two animals were “body arching laterally” still observed during the third minute of bleeding. These two animals also expressed signs of reduced stunning effectiveness, i.e., respiratory movements and/or tongue tone. However, based on these two single cases, we would not recommend late “body arching laterally” as a sole indicator of an inadequate stunning effect, but the occurrence of this movement after the end of the second minute of bleeding should lead to intensive monitoring for signs of an active brainstem.

An association between stunning effectiveness and movements cannot be statistically proven in this study due to the small number of animals with reduced stunning effectiveness. However, when looking at the movements monitored and scores calculated in cattle showing signs of shallow depth of stunning, these are comparable to those of properly stunned cattle when analysed on a case-by-case analysis.

Based on these results, the authors would like to emphasise the importance of paying attention to reliable indicators such as eye movements and/or resumption of breathing to evaluate stunning effectiveness, and not to be distracted by, e.g., kicking movements of the limbs or lateral body arching. The indicators have to be assessed in context and not just considered individually. The results show that movements occur regularly in properly stunned cattle.

### 4.5. Limitations

Due to technical reasons, we could not precisely measure the efficiency of the exsanguination, which could have had an effect either on stunning effectiveness or on movements. However, all staff involved were experienced and promptly repeated sticking if reduced blood flow was suspected. Another possible limitation in this study is that the values for the key parameters of bolt exit length and bolt velocity could only be approached and thus were not measured for every single shot. Currently, there is no technical solution to measure bolt velocity during the shot. Regarding the bolt exit length, examining a section of the skull of every animal would have been a possible solution, but this could not be included because of limitations in resources. As stated above, due to the small number of animals showing signs of reduced stunning effectiveness, only case-by-case analysis was possible concerning an association between stunning effectiveness and movements. Nevertheless, considering the current state of scientific knowledge, we assume that indicators other than movement are far more important for evaluating the effectiveness of stunning in cattle after captive bolt stunning.

### 4.6. Animal Welfare Implications

This description and analysis of movements can contribute to assessing the stunning effectiveness of cattle after captive bolt stunning more reliably. According to our results, most movements observed are not suitable to determine an insufficient stunning effect. When assessing stunning efficiency, we recommend looking for signs at the head such as breathing, eye movements, or recurrent ear tension since other movements like leg and body movements can still occur a few minutes after sticking in well-stunned cattle. The identification of the factors influencing these movements can contribute to understanding the movements, optimising the stunning process, and the further development of stunning devices. In order to correctly assess movements, the staff responsible for slaughter should be able to distinguish between conscious righting attempts and unconscious body or limb movements. Impressive movements after stunning should not divert the attention of those responsible for detecting the pertinent signs of reduced stunning effectiveness, which are comparatively inconspicuous. On the other hand, movements can have an impact on animal welfare when they impede prompt shackling and sticking, thereby posing challenges for employees in efficiently and safely performing these tasks. This aspect must be considered, as staff play a key role in maintaining high animal welfare standards during slaughter. Therefore, it is necessary to continue looking for ways to reduce movements.

## 5. Conclusions

For the first time, movements in cattle after captive bolt stunning were categorised and systematically described from landing up to at least the end of the fourth minute of bleeding. A total of 93.4% of all cattle examined showed at least one movement within the observation period. Although most cattle movements ended one minute after sticking, there were individual animals that showed movements for even longer, sometimes up to eight minutes after sticking. Factors affecting the category and frequency of movements could be identified, in relation to both the animals and the features of the captive bolt devices used. Breed, sex category, and the exit length and function type of the stunning device were the main impact factors found. The common field experience of Black and Red Holstein cows showing especially strong movements during landing and hoisting was confirmed. In total, 48.9% of all cows showed movements on the landing grid, whereas related to the whole observation period, bulls and heifers had a higher movement activity (sum score) than cows. Factors related to the stunning device also had an impact on movements. Both the use of pneumatically powered devices and the use of captive bolt guns with an increased exit length resulted in a significantly lower sum score. However, the variables investigated explain only 12% of the variance; thus, the effects of unknown confounding variables cannot be excluded.

Excitatory movements at landing hinder the ability of employees to quickly and safely perform hoisting and sticking. As the occurrence and intensity of movements are only explained to a limited extent by the identified process-related factors, it is necessary to continue looking for ways to reduce movements from landing to sticking.

Out of 2911 cattle, 99.4% showed no signs of reduced stunning effectiveness. This positive development in enhanced stunning effectiveness is attributed to, among other things, the use of modern stunning devices (esp. pneumatic-driven stunners) and stun boxes with tight head restraint. Thus, slight angular or positional shooting deviations do not necessarily lead to a reduced stunning effect. Movements of limbs or the tail, as well as lateral or ventral movements of the head and/or trunk, do not indicate limited stunning effectiveness. Only “body arching laterally” still being expressed in the third minute of bleeding might be an indication for insufficient stunning and bleeding, but it should only be considered together with respiratory movements or preserved eye reflexes. It is assumed that after successful captive bolt stunning in cattle, movements commonly occur for several minutes after the shot and, with the exception of “arching backwards”, are not a suitable sole indicator of reduced stunning effectiveness. In terms of animal welfare, it is crucial to accurately recognize signs of reduced stunning effectiveness. Staff, veterinarians, and external inspectors should prioritize signs of an active brainstem over limb, body, and tail movements. Ensuring the correct identification of signs of remaining or regaining consciousness is a key factor in guaranteeing animal welfare at slaughter.

## Figures and Tables

**Table 1:** Features for restraining, stunning, and bleeding at the participating abattoirs.

Abattoir	Approximate Slaughter Capacity per Day (no. of Cattle)	Slaughter Speed per Hour (no. of Cattle/h)	Number of Staff Performing the Stunning	Stunning Device	Head Restraint/Manufacturer	Mean Stun-to-Stick Interval (s)	Bleeding Time before further Processing (min)
A	400	50	2	EFA^®^ VB 315 (Schmid & Wezel GmbH, Maulbronn, Germany); Schermer^®^ KR, KS (Freund Maschinenfabrik GmbH & Co. KG, Paderborn, Germany)	Tight restraint ^1^, Allkon^®^ (Allkon GmbH & Co. KG, Steinau an der Straße, Germany)	37.8	3.0
B	700	65	3	Jarvis^®^ USSS-21 (Jarvis GmbH, Buchholz, Germany)	Tight restraint ^1^, MPS^®^ (Marel, Lichtenvoorde, Netherlands)	47.5	7.0
C	1000	60–70	3	EFA^®^ VB 315 (Schmid & Wezel GmbH, Maulbronn, Germany);	Tight restraint ^1^, BANSS^®^ (JWE-BANSS GmbH; Biedenkopf, Germany)	49.6	6.0
D	500	55	4	EFA^®^ VB 315 (Schmid & Wezel GmbH, Maulbronn, Germany);	Tight restraint ^1^, DGS^®^ (DGS Processing Solution, Haaksbergen, Netherlands)	42.0	6.0
E	350	50–55	2	Schermer^®^ KL, KS (Freund Maschinenfabrik GmbH & Co. KG, Paderborn, Germany)	Loose (passive), self-built	51.0	4.5

^1^ Tight restraint: lateral and vertical restriction of movement of the head [2].

**Table 2:** Specifications of captive bolt stunners used, including key parameters according to Reg. (EC) No. 1099/2009 [2].

Abattoir	Manufacturer, Type of Stunning Device	Function Type of Stunning Device	Pressure ^1^ or Cartridge Strength Used	Velocity in Air in (m/s) ^4^	Extension Lengths in (mm) ^4^	Diameter in (mm) ^4^	Kinetic Energy in (Joule)	Position of the Person in Charge of Stunning
A	EFA^®^ VB 315 (Schmid & Wezel GmbH, Maulbronn, Germany)	Pneumatic	13.5 bar	40.1	119.0	14.5	453	Left of the animal
A	Schermer^®^ KR (Freund Maschinenfabrik GmbH & Co. KG, Paderborn, Germany)	Cartridge	Red ^2^	58.3	88.0	12.0	389	Left of the animal
A	Schermer^®^ KS (Freund Maschinenfabrik GmbH & Co. KG, Paderborn, Germany)	Cartridge	Red ^2^	52.6	81.0	12.0	334	Left of the animal
B	Jarvis^®^ USSS-21 (Jarvis GmbH, Buchholz, Germany)	Pneumatic	15.5 bar	43.5	88.0	12.0	488	Right of the animal
B	Jarvis^®^ USSS-21 (Jarvis GmbH, Buchholz, Germany)	Pneumatic	15.5 bar	37.6	90.0	14.5	421	Right of the animal
C	EFA^®^ VB 315 (Schmid & Wezel GmbH, Maulbronn, Germany)	Pneumatic	14.2 bar	41.6	119.0	14.5	488	Left of the animal
D	EFA^®^ VB 315 (Schmid & Wezel GmbH, Maulbronn, Germany)	Pneumatic	14.2 bar	41.6	119.0	14.5	488	Front of the animal
E	Schermer^®^ KL (Freund Maschinenfabrik GmbH & Co. KG, Paderborn, Germany)	Cartridge	Red ^2^	58.4	121.0	12.0	433	Right of the animal
E	Schermer^®^ KS (Freund Maschinenfabrik GmbH & Co. KG, Paderborn, Germany)	Cartridge	Blue ^3^	50.3	79.0	12.0	306	Right of the animal

^1^ Air pressure as shown by the manometer; ^2^ red cartridge strength corresponds to 4.9 grain or 320 mg; ^3^ blue cartridge strength corresponds to 4.8 grain or 315 mg; and ^4^ key parameters according to Reg. (EC) No. 1099/2009.

**Table 3:** The evaluation scheme for stunning effectiveness is mainly based on EFSA AHAW Panel [23], von Holleben et al. [24], and von Wenzlawowicz et al. [9].

Body Part	Doubtful Effect of Stunning ^1^		Insufficient Stunning Effect ^2^	
	Parameter	Definition	Parameter	Definition
Whole body	Abnormal cramps	The animal does not show a typical tonic or tonic–clonic convulsive phase after the shot [10]	No collapse after shot, posture	Failure to collapse, attempts to regain posture after being shot
			Arched back righting reflex	Arching of the back and sustained backward lifting of the head, while the animal hangs on the rail [25]; the symptom can also be shown while an animal is lying in a horizontal position [26]
Ears			Ear tone	Ears do not hang down limply but are tense or straightened (visual and palpatory examination in case of suspicion)
Eyes	Eyeball rotation	The eyeball is not centred within 25 s after the shot; the eyeballs may be rotated to a great extent so that the pupils may not be visible [10]; the eyeball is rotated [27]	Corneal reflex	Repeated blinking response elicited by touching or tapping the cornea
	No pupil dilatation	The pupil is not fully dilated	Spontaneous blinking	Spontaneous closing of the eyelids without prior irritation of the eyelid or cornea
	Nystagmus	Spontaneous rapid side-to-side movements of the eyeball	Focused eye movements	Accommodation of the eye, the eyeball follows movements in the vicinity
	Corneal reflex	Single blinking response elicited by touching or tapping the cornea		
Respiratory system	Breathing movements (<4 times)	The animal shows up to 3 breathing movements after the shot, which can be recognised by movements of the flank, muzzle, or nostrils	Breathing movements (≥4 times)	The animal shows more than 3 breathing movements after the shot, which can be recognised by movements of the flank, mouth, or nostrils
			Vocalisation	Vocalisation in the form of moaning, grunting, or mooing

^1^ Transition zone between definitely unconscious/brain dead and definitely conscious but low risk of awakening; no pain and suffering but first indicators for a shallow depth of stunning [25]; ^2^ high risk of regaining consciousness (still in transition zone) as well as remaining or regained consciousness. Signs of a conscious state are no loss of posture, righting reflex, spontaneous blinking, and focused eye movements.

**Table 4:** Definitions of post-stun movement categories and scores (for examples, see also the Supplementary Materials).

Movement Category	Definition	Intensity ^1^	Severity of Movement, Score Explanation	Score Value
Kicking hind limb	A single/free hind limb is moved away from the body and back repeatedly and rapidly.	Moderate ^2^	Fast Vigorous Dangerous to workers	2.0
		Intense ^3^	Very fast Vigorous Dangerous to workers	3.0
Twitching	More than one limb is moving, possibly together with the trunk and neck. The movement can be synchronous or asynchronous and may involve the hind limb and forelimb (e.g., forelimb paddling together with hind limb kicking).	Moderate ^2^	Fast Vigorous Dangerous to workers	2.0
		Intense ^3^	Very fast Vigorous Dangerous to workers	3.0
Bending forelimb	One or both forelimbs are bent towards the body. The movement can be synchronous or asynchronous, single or repeated (e.g., forelimb paddling with bent limbs).		Either fast or slow Predominantly vigorous May impede the work	1.0
Lifting forelimb	One or both forelimbs lift in an extended position. The movement can be synchronous or asynchronous, single or repeated (e.g., forelimb paddling with stretched limbs).		Either fast or slow Predominantly vigorous May impede the work	1.0
Body arching laterally	The longitudinal axis of the hoisted animal is bending to one side and the head and body are not hanging straight down; in most cases without repetition.		Either fast or slow Predominantly vigorous May impede the work	1.0
Body arching ventrally	The head and possibly the trunk of the hoisted animal are bending ventrally; in most cases without repetition.		Either fast or slow Predominantly vigorous May impede the work	1.0
Stretching hind limb	The free hind limb extends away from the body for at least 3 s.		Slow No repetition within 5 s No impact on work	0.5
Bending hind limb	The free hind leg is bent and pulled towards the body.		Slow No repetition within 5 s No impact on work	0.5
Arching backwards/righting reflex	Arching of the back and sustained backward lifting of the head, while the animal hangs on the rail [25].		Slow No repetition within 5 s No impact on work	0.5
No movement	Animal shows none of the movements listed above.			0

^1^ A graduation of intensities is only used for kicking and twitching during LANDING and HOISTING: from the process interval SKIN INCISION onwards, no differentiation was made between moderate and intense; ^2^ Moderate: 1. frequency <1/s and duration <5 s; 2. frequency >1/s and duration <5 s; and 3. frequency <1/s and duration >5 s; ^3^ Intense: frequency >1/s and duration >5 s; example for the calculation of the sum score: An animal shows “kicking hind limb intense” (3.0) at LANDING, “body arching ventrally” (1.0) at STICKING, “kicking hind limb” (2.0) and “bending forelimb” (1.0) in the FIRST MINUTE OF BLEEDING and “stretching hind limb” (0.5) in the FOURTH MINUTE OF BLEEDING. The scores for the individual process intervals would then look as follows: LANDING 3.0, HOISTING 0, SKIN INCISION 0, STICKING 1.0, FIRST MINUTE OF BLEEDING 3.0 (2.0 + 1.0), SECOND MINUTE OF BLEEDING 0, THIRD MINUTE OF BLEEDING 0, and FOURTH MINUTE OF BLEEDING 0.5. This results in a total sum score of 3.0 + 0 + 0 + 1.0 + 3.0 + 0 + 0 + 0.5 = 7.5.

**Table 5:** Movement categories are shown by cattle following stunning across various breeds (relative frequency).

Breed Type	Kicking HL/Kicking HL Moderate	Kicking HL Intense	Twitching/Twitching Moderate	Twitching Intense	Stretching HL	Body Arching Laterally	Body Arching Ventrally	Bending FL	Lifting FL	Bending HL
Brown Swiss	53.0%	2.0%	9.0%	0.0%	28.0%	7.0%	21.0%	21.0%	45.0%	5.0%
Charolais	71.1%	11.1%	15.6%	15.6%	42.2%	11.1%	37.8%	53.3%	62.2%	6.7%
German Angus	71.4%	7.1%	42.9%	21.4%	33.3%	19.0%	40.5%	40.5%	57.1%	2.4%
German Red Pied	76.1%	11.9%	14.9%	9.0%	38.8%	14.9%	25.4%	43.3%	56.7%	6.0%
Simmental	58.5%	4.8%	10.4%	3.5%	39.9%	13.4%	25.9%	23.8%	44.7%	4.7%
Limousin	64.4%	16.7%	13.3%	18.9%	50.0%	10.0%	35.6%	35.6%	66.7%	2.2%
Red Holstein	54.9%	16.0%	16.0%	19.4%	23.3%	7.8%	22.3%	46.6%	57.8%	2.9%
Black Holstein	57.6%	11.7%	20.9%	20.6%	26.0%	8.3%	15.2%	43.8%	35.2%	3.1%
CBB	69.6%	4.0%	16.0%	9.6%	46.4%	12.0%	28.0%	35.2%	69.6%	5.6%
CBD	65.3%	8.0%	23.1%	6.4%	39.6%	11.1%	32.1%	38.7%	57.5%	2.4%
Total	60.2%	9.6%	18.1%	13.5%	32.9%	10.2%	22.7%	38.1%	46.2%	3.5%

FL = forelimb; HL = hind limb; CBB = crossbreed beef × beef; CBD = crossbreed beef × dairy.

**Table 6:** The results of the contingency test: relative frequency of movement categories by process interval and stunning device function type (pneumatic and cartridge).

Category of Movement	Landing		Hoisting		Skin Incision		Sticking		1st min. of Bleeding		2nd min. of Bleeding		3rd min. of Bleeding		4th min. of Bleeding	
	P	C	P	C	P	C	P	C	P	C	P	C	P	C	P	C
Kicking HL; incl. moderate	7.4 *	11.2 *	11.3 *	19.8 *	12.7	11.6	27.1	24.9	26.6 *	38.6 *	2.2	1.9	1.0	0.8	3.2	5.1
Kicking HL intense	2.4 *	6.0 *	4.4 *	13.3 *												
Twitching incl. moderate	9.9 *	5.5 *	10.5 *	5.6 *	0.6	0.7	0.2	0.3	0.1	0.0	0.0	0.0	0.0	0.0	0.0	0.0
Twitching intense	10.4	13.7	2.5	2.2												
Stretching HL	0.1	0.3	5.8 *	9.8 *	3.2	3.0	3.9	4.1	17.2	15.3	1.9	1.5	2.3	1.8	6.7	8.6
Body arching laterally	0.0	0.0	4.9	3.0	3.5	2.9	2.5	3.4	0.2 *	2.7 *	0.1	0.1	0.0	0.0	0.0	0.0
Body arching ventrally	0.0	0.0	0.2	0.0	8.7 *	2.7 *	17.8	20.0	0.9	2.1	0.0	0.0	0.1	0.0	0.0	0.0
Bending FL	0.0	0.0	7.0	8.6	9.2	12.3	10.7 *	17.2 *	16.0 *	22.0 *	5.9	6.4	3.2	1.6	1.3	0.4
Lifting FL	0.0	0.0	1.5 *	3.1 *	8.3 *	14.0 *	11.6 *	17.6 *	28.2 *	36.1 *	4.4	4.8	1.0	0.0	0.7	0.5
Bending HL	0.0	0.0	0.7	1.0	0.7	0.3	0.3	0.8	0.6	0.7	0.4	0.1	0.5	0.3	0.5	0.4

P = pneumatically powered device; C = cartridge-powered device; FL = forelimb; HL = hind limb; * high statistical difference between function types (*p* < 0.001).

## Data Availability

The data are not publicly accessible due to security concerns for the companies involved. Further enquiries can be directed to the author.

## Supplementary Materials

With regard only to the definition of movements, the following supporting information (video examples) can be downloaded at: https://doi.org/10.5281/zenodo.10572807 (accessed on 19 March 2024), Video S1: 1 Kicking hind limb moderate landing unconscious cow; Video S2: 2 Twitching intense landing unconscious cow; Video S3: 3 Kicking hind limb intense landing unconscious cow; Video S4: 4 Twitching moderate hoisting unconscious bull; Video S5: 5 Twitching intense hoisting unconscious bull; Video S6: 6 Body arching laterally hoisting unconscious heifer; Video S7: 7 Stretching hind limb hoisting lifting forelimb sticking unconscious bull; Video S8: 8 Body arching ventrally kicking hind limb sticking 1st min of bleeding unconscious bull; Video S9: 9 Body arching laterally 1st min of bleeding unconscious bull; Video S10: 10 Body arching ventrally bending hind and forelimbs 1st min of bleeding unconscious heifer; Video S11: 11 Lifting forelimb kicking hind limb 1st min of bleeding unconscious bull; Video S12: 12 Bending hind limb 3rd minute of bleeding unconscious bull; Video S13: 13 Bending forelimb bending hind limb 4th minute of bleeding unconscious cow; Video S14: 14 Stretching hind limb with shivering sixth min of bleeding unconscious cow; Figure S1: 15 Arching backwards.
